# Do Feelings of Defeat and Entrapment Change over Time? An Investigation of the Integrated Motivational—Volitional Model of Suicidal Behaviour Using Ecological Momentary Assessments

**DOI:** 10.3390/ijerph17134685

**Published:** 2020-06-29

**Authors:** Jana-Sophie Stenzel, Inken Höller, Dajana Rath, Nina Hallensleben, Lena Spangenberg, Heide Glaesmer, Thomas Forkmann

**Affiliations:** 1 Institute of Experimental Psychology, Heinrich-Heine University Düsseldorf, Düsseldorf, 40225 Düsseldorf, Germany; jana-sophie.stenzel@uni-duesseldorf.de; 2 Department of Clinical Psychology, University of Duisburg-Essen, 45141 Essen, Germany; inken.hoeller@uni-due.de (I.H.); dajana.rath@uni-due.de (D.R.); 3 Department of Medical Psychology, University of Leipzig, 4109 Leipzig, Germany; nina.hallensleben@medizin.uni-leipzig.de (N.H.); lena.spangenberg@medizin.uni-leipzig.de (L.S.); heide.glaesmer@medizin.uni-leipzig.de (H.G.)

**Keywords:** defeat, entrapment, suicide, suicidal ideation, ecological momentary assessment, integrated motivational–volitional model of suicidal behavior

## Abstract

(1) Background. Defeat and entrapment have been highlighted as major risk factors of suicidal ideation and behavior. Nevertheless, little is known about their short-term variability and their longitudinal association in real-time. Therefore, this study aims to investigate whether defeat and entrapment change over time and whether defeat predicts entrapment as stated by the integrated motivational–volitional model of suicidal behavior. (2) Methods. Healthy participants (*n* = 61) underwent a 7-day smartphone-based ecological momentary assessment (EMA) on suicidal ideation/behavior and relevant risk factors, including defeat and entrapment and a comprehensive baseline (T0) and post (T2) assessment. (3) Results. Mean squared successive differences (MSSD) and intraclass correlations (ICC) support the temporal instability as well as within-person variability of defeat and entrapment. Multilevel analyses revealed that during EMA, defeat was positively associated with entrapment at the same measurement. However, defeat could not predict entrapment to the next measurement (approximately two hours later). (4) Conclusion. This study provides evidence on the short-term variability of defeat and entrapment highlighting that repeated measurement of defeat and entrapment—preferably in real time—is necessary in order to adequately capture the actual empirical relations of these variables and not to overlook significant within-person variability. Further research—especially within clinical samples—seems warranted.

## 1. Introduction

According to the WHO [1], approximately 800,000 people worldwide die by suicide each year; however, the number of nonfatal attempts is estimated to potentially be 20 times higher. While people of all age groups die by suicide, it is the second leading cause of death among 15–29-year-olds. In order to improve prevention of fatal as well as nonfatal suicide attempts, it is important to research risk factors such as suicidal ideation.

Studies suggest that possibly 1/3 of people could face suicidal ideation in the course of their lifetime [2]. In a representative sample of the German general population, 8% of the participants reported suicidal ideation within the past two weeks [3]. These numbers seem alarming, especially because the transition from suicidal ideation to suicide attempt appears to happen quickly [4].

One contemporary theoretical model aiming at explaining the development of suicidal ideation and the transition to suicidal behavior is the integrated motivational–volitional model of suicidal behavior (IMV model, [5]). Its current, slightly revised version was published by O’Connor and Kirtley in 2018 [6]. The model integrates constructs of previous older models (e.g., the cry of pain model [7] and the interpersonal theory of suicidal behavior [8]) into one cohesive model of suicidal behavior and describes the development of suicidal behavior within three phases (similar to the theory of planned behavior; [9]). The first phase of the model is the premotivational phase, which focuses on background factors and triggering events. The second phase of the model, the motivational phase, describes the process of suicidal ideation and intention formation. Core components of this phase are defeat and entrapment, which eventually lead to suicidal ideation and intent. The relationship between defeat and entrapment is hereby moderated by the so-called threat to self-moderators (e.g., social problem-solving, coping, memory biases). The relationship between entrapment and suicidal ideation is moderated by motivational moderators (e.g., thwarted belongingness, perceived burdensomeness, social support). Lastly, the volitional phase describes the process of the behavioral enaction of suicidal behavior. Volitional moderators (e.g., access to means, exposure to suicide, fearlessness about death) hereby moderate the relationship between suicidal ideation and suicidal behavior. Additionally, a feedback loop exists between suicidal ideation/intent and suicidal behavior.

The model provides empirically testable hypotheses. Furthermore, it tries to account for the complexity of suicidal behavior and allows distinguishing between suicidal ideation and suicidal behavior. A number of studies underline the general validity of the model (e.g., [10,11,12]). Similarly, previous studies have shown that defeat and entrapment are core components on the pathway to the formation of suicidal ideation and transdiagnostically important constructs (e.g., [13,14]).

Defeat and entrapment are both core components of the social rank theory [15]. Defeat is mostly defined as feeling powerless or defeated by life, often accompanied by a loss of social status and the missing of personal goals. Entrapment, on the other hand, relates to the feeling of being entrapped in a situation with no possibility to escape from or to cope with internal and/or external stressors. While there has been some debate on whether defeat and entrapment are actually two different constructs (e.g., [16,17,18,19]), a recent study could show that—despite their autocorrelation—they are in fact two distinct constructs [20]. In a first longitudinal study including defeat and entrapment, Taylor, Gooding, Wood, Johnson, and Tarrier [21] found that 50% of variance accounted for within-person variance for both constructs in 79 students who reported suicidality during a 12-month follow-up study. In another 12-month follow-up study, Griffiths, Wood, Maltby, Taylor, and Tai [18] found that variance in defeat and entrapment was predominantly due to between-person fluctuations. On the other hand, in a daily diary study assessing young adults with nonsuicidal self-injury disorder, Cloos, Di Simplicio, Hammerle, and Steil [22] could find variance in entrapment, which was predominantly due to within-person fluctuations. Regarding the core assumptions of the IMV model, studies could find that entrapment mediated the effect of defeat on suicidal ideation (e.g., [23]). However, none of these studies examined potential changes in defeat and entrapment over time using short follow-up intervals of minutes or hours. Thus, although within-person variance in defeat and entrapment has been shown, it remains unclear whether the two constructs change over short time intervals.

A beneficial methodological approach to measure self-reported thoughts and behavior repeatedly over short time intervals is smartphone-based ecological momentary assessment (EMA). EMA is defined as the repeated sampling of current behavior and current experiences in real time within the natural environment of participants (e.g., via smartphone; [24]). Benefits of EMA include no/less memory bias, higher ecological validity, and better compliance (e.g., [25,26,27]). Additionally, EMA allows examining dynamic constructs in short/moment by moment time frames. It shall be noted that while some researchers might fear that repeatedly assessing suicidal ideation and related risk factors might increase suicidal ideation, DeCou and Schumann [28] could find no such effect in a recent meta-analysis. Feasibility of applying EMA for the assessment of suicidal ideation and related risk factors has been demonstrated [25].

Although its potential methodological benefits for the field of suicidology have already been recognized some years ago [29,30], only very few EMA studies exist so far (e.g., [31]). Only recently could research using EMA show that suicidal ideation fluctuates substantially (e.g., [32,33]). In an EMA study, Hallensleben and colleagues [34] found that 25% and 36% of variance in passive and active suicidal ideation, respectively, was within-person variability. Similar results were reported by Kleiman and colleagues [33]. In line with these findings, proximal risk factors of suicide also seem to be subject to substantial fluctuations (e.g., [34,35]). For instance, Hallensleben and colleagues [34] and Spangenberg and colleagues [36] could show that thwarted belongingness (TB), perceived burdensomeness (PB), and capability for suicide (CS)—which are considered to be risk factors for suicidal ideation within the IMV model [5,6] and the interpersonal theory of suicide (IPTS, [8])—fluctuate as well and prospectively predict suicidal ideation. Overall, these studies show that suicidal ideation and associated predictors seem to be subject to fluctuations over short intervals. Thus, assessing suicidal ideation and behavior and potentially associated risk factors repeatedly over short time intervals appears to be mandatory in order to improve our understanding of the occurrence and time course of suicidal ideation and behavior.

Even though defeat and entrapment are supposed to be key mechanisms for suicidal ideation and suicidal behavior and form the central and direct pathway to suicidal ideation within the IMV model, fluctuation over time of these constructs has never been examined before using EMA. Additionally, little is known about the prevalence of feelings of defeat and entrapment in healthy individuals, as most previous studies focused on patient or mixed samples (e.g., [20]). Therefore, the present study aims to investigate whether feelings of defeat and entrapment change over time in healthy participants. Additionally, we aim to examine the relationship between defeat and entrapment as proposed by the IMV model [6]. The following hypotheses were tested empirically in this study:

**Hypothesis** **1a (H1a):** Feelings of defeat and entrapment change over time in healthy participants when comparing a baseline (T0) with a post (T2) assessment eight days later. Therefore, we expect significant differences in defeat and entrapment between both assessment points of at least small size.

**Hypothesis** **1b (H1b):** Feelings of defeat and entrapment change over time in healthy participants during a 7-day EMA. Therefore, we expect considerable within- as well as between-person variability.

**Hypothesis** **2a (H2a):** During a 7-day EMA of healthy participants, defeat predicts entrapment cross-sectionally (at the same measurement) as proposed by the IMV model [6]. Thus, we expect a significant positive relationship between defeat and entrapment at the same measurement.

**Hypothesis** **2b (H2b):** During a 7-day EMA of healthy participants, defeat predicts entrapment prospectively (from measurement to measurement) as proposed by the IMV model [6]. Therefore, we expect a significant positive relationship between defeat and entrapment from measurement to measurement, even when controlling for autocorrelative effects of entrapment.

## 2. Materials and Methods

### 2.1. Sample

The entire sample consisted of *n* = 61 participants. Participants were between 18 and 51 years old (*M* = 24.21, *SD* = 6.99). Fifty-four participants were women (88.5%). Twenty participants reported a mental illness in their past (32.8%; assessed via a structured clinical interview (Mini-DIPS, [37,38]); see baseline (T0) assessment).

Participants had to be at least 18 years old. They needed to have sufficient knowledge of the German language and normal or corrected to normal eyesight. Furthermore, participants should not be mentally ill and should not abuse alcohol (>20/40 g of alcohol daily) or use drugs. Participants were randomly assigned to one of two groups. A two-group design was used to examine different facets of interoception over time with one group (*n* = 31) completing interoception tasks within EMA, while the other group was not (*n* = 30). This part of the study is reported elsewhere [39]. To keep the sample comparable to other studies assessing interoception (e.g., [40]), participants needed to be physically healthy with a body mass index (BMI) between 18.5 and 24, without participating in competitive or endurance sports more than three times a week, and without medication influencing the cardiovascular system.

Once participants declared their interest via email, they were called and interviewed regarding the inclusion criteria mentioned above. A total of 176 participants underwent the telephone interview. Ninety-five participants were excluded from further assessments because they had no time for appointments or did not meet the inclusion criteria of the study. A total of 81 participants fulfilled the inclusion criteria of the study and were invited for further testing.

**Figure 1:**
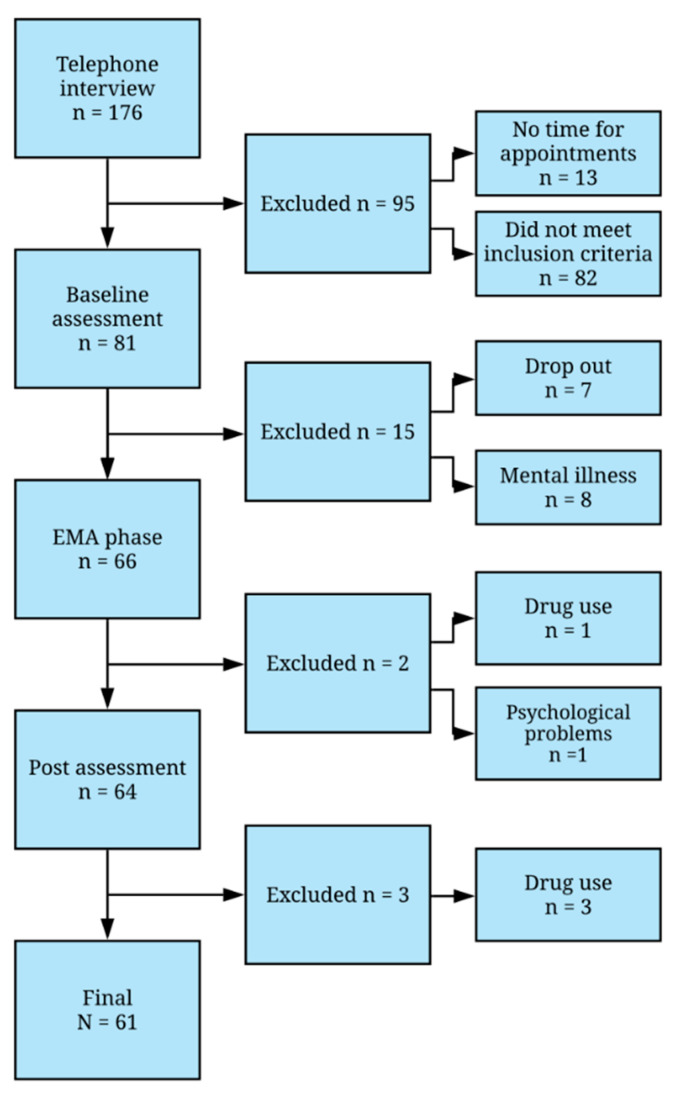
Flowchart of study inclusion. EMA = ecological momentary assessment.

Another 20 participants were lost during the course of the study due to general drop-out or were excluded because they reported drug use or mental illnesses at later stages of the assessment. Figure 1 shows the recruitment process and study flow, including the final number of participants.

The study followed the ethical guidelines of the Declaration of Helsinki [41] and was approved by the ethics committee of the Institute of Psychology of the University Duisburg-Essen (ethics vote from 15 May 2019). Participants received 60 € or five hours of study credit and 10 € if they completed the entire study. All participants gave written informed consent prior to participation.

### 2.2. Measures and Procedure

**Figure 2:**
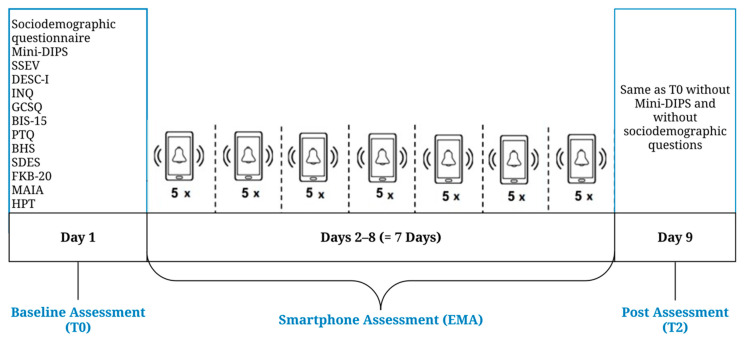
Study design. Mini-DIPS = structured clinical interview for diagnosing mental disorders according to ICD-10; SSEV = suicidal ideation and suicidal behavior scale [42]; DESC-I = Rasch-based depression screening, Version 1 [43,44]; INQ = interpersonal needs questionnaire ([45]; German version: [46]); GCSQ = German Ccpability for suicide questionnaire [47]; BIS-15 = Barratt impulsiveness scale ([48]; German version: [49]); PTQ = perseverative thinking questionnaire [50]; BHS = Beck hopelessness scale ([51]; German version: [52]); SDES = short defeat and entrapment scale; FKB-20 = Fragebogen zum Körperbild (German body image questionnaire; [53]); MAIA = multidimensional assessment of interoceptive awareness ([54]; German version: [55]); HPT = heartbeat perception task [56]; EMA = ecological momentary assessment.

Assessments took place between June and December 2019. Figure 2 provides an overview of the study procedure.

#### 2.2.1. Baseline Assessment (T0)

The baseline assessment (T0) took place at the Department for Clinical Psychology at the Institute of Psychology at the University of Duisburg-Essen and lasted approximately 1.5 h.

First, participants took part in a structured clinical interview (Mini-DIPS, [37,38]) to diagnose mental illnesses. If participants were diagnosed with a current mental illness, they were excluded from further assessment.

A self-designed sociodemographic questionnaire was used to assess sociodemographic information. All eligible participants also completed ten questionnaires, assessing various theoretical constructs related to suicidal ideation and behavior (see Figure 2). Especially relevant to the current study was the assessment of defeat and entrapment.

Defeat and entrapment were assessed using the short defeat and entrapment scale (SDES, [19]; German version: [57]), with four items assessing defeat on a five-point Likert scale from “never” (0) to “always” (4) and four items assessing entrapment on a five-point Likert scale from “not at all” (0) to “very strongly” (4). Higher scores indicate higher levels of defeat and entrapment. Cronbach’s alpha [58] was good at both points of assessment (Defeat: α_(T0)_ = 0.83 and α_(T2)_ = 0.85; Entrapment: α_(T0)_ = 0.77 and α_(T2)_ = 0.78).

Lastly, interoceptive accuracy and awareness were assessed via electrocardiogram (ECG) and the heartbeat perception task (HPT, [56]). As mentioned above, the assessment of interoception is not relevant to the current study and will be reported elsewhere [39].

At the end of T0, all participants received a briefing on smartphone-based assessment procedures (i.e., overview over the app, charging the phone, carrying the phone at all times). All participants received Android smartphones (Samsung or Motorola) for the duration of the EMA.

#### 2.2.2. Ecological Momentary Assessment (EMA)

Starting the day after the baseline (T0) assessment, participants underwent a 7-day EMA consisting of five assessments per day, resulting in a maximum of 35 assessments per participant. All data collection was conducted via movisensXS (Version 0.7.4162). Assessments were randomly assigned between 8 a.m. and 8 p.m. with at least two hours in between each assessment. Participants could postpone or completely reject a prompt if they were not able to answer. When the phone battery ran below 20%, participants received a notification to charge the phone. Since single assessments were short, the overall time of EMA was <30 min per day.

EMA items assessed momentary ratings of depression (two items), positive affect (two items), defeat (two items), entrapment (two items), passive and active suicidal ideation (two items each; four items total), capability for suicide (three items), and context variables (four items).

Most EMA items were part of an EMA item set previously used by the research group (e.g., [34]). The item set proved to be feasible in previous studies and showed high reliability as well as convergent validity [25]. Defeat items were taken from the defeat scale ([15]; German version: [59]) and entrapment items from the entrapment scale ([15]; German version: [60]). Items were selected with regard to factor loadings [15,60] as well as item content and wording. Wording was adjusted to optimally relate to the actual moment. Sequencing of items was kept constant at each measurement.

Individual results were uploaded from the smartphone to a webserver directly after one assessment was completed, allowing the research team to check compliance rates. Researchers could also send messages to participants. Each participant received up to three text messages for motivational purposes, to answer technical questions or in case their compliance rate dropped below 80%.

#### 2.2.3. Post Assessment (T2)

The post (T2) assessment took place no longer than seven days after the EMA at the Department for Clinical Psychology at the Institute of Psychology at the University of Duisburg-Essen and lasted approximately one hour. The post (T2) assessment was identical to the baseline (T0) assessment, except that participants did not have to complete the Mini-DIPS and the sociodemographic questionnaire.

### 2.3. Statistical Analysis

#### 2.3.1. Variability of Defeat and Entrapment (Hypotheses 1a and 1b)

In a first step, *t*-tests for dependent samples and effect sizes *d* [58] including 95% confidence intervals (CI) were calculated to test whether feelings of defeat and entrapment differed between baseline (T0) and post (T2) assessment (Hypothesis 1a). For statistical analyses, IBM SPSS Statistics (Version 26.0) was used.

Second, changes in feelings of defeat and entrapment during EMA were analyzed to test whether feelings of defeat and entrapment change over time during EMA (Hypothesis 1b). Data consisted of 35 (assessments at level one) × 61 (persons at level two) = 2135 observations. With 82.2% of completed EMA, a total of 1755 valid observations were recorded. Sufficient observations were recorded to detect moderate to large effects [61]. Intercept-only models were calculated for both defeat and entrapment using R [62] and the packages car [63], lme4 [64], lmerTest [65], and effects [63]. Additionally, intraclass correlations (ICC; [66]) were calculated to examine the proportion of variance explained by the two different levels. Point-to-point variability was assessed calculating mean squared successive differences (MSSD, [67]), using the psych [68] package. Results were visualized by creating heatmaps. Figures were produced using the package 2 (version 3.2.1; [69]). As a rule of thumb, simple two-level models should evaluate at least 30 units per level [70]. In order to achieve equal and adequately powered sample sizes for both of our subgroups (interoception vs. no interoception; see inclusion criteria), we had aimed for 30 participants per subgroup, resulting in a planned sample size of at least *n* = 60 participants overall.

#### 2.3.2. Relationship between Defeat and Entrapment According to the IMV Model (Hypotheses 2a and 2b)

In a third step, the question of whether defeat predicts entrapment as stated by the IMV model [6] was examined by testing associations between defeat and entrapment cross-sectionally (at the same measurement, hypothesis (2a) as well as prospectively (from measurement to measurement, hypothesis (2b). The intercept-only model for entrapment was used as a baseline model (M0). Quasi R^2^ [71] was used to indicate changes of the residual variance in entrapment when adding the model’s additional level 1 predictors [66]. For statistical analyses—regarding the comparison between baseline and fitted models—HLM (Version 7.01) was used. A total of six models were fitted. All models were estimated by means of restricted maximum likelihood estimation (REML), since the number of level 2 units is small [72]. The level 1 predictor variables were person-mean-centered in all analyses since we were mostly interested in within-person relationships [73]. In order to test hypothesis 2a (cross-sectional), models 1 and 2 contained defeat at *t* at level 1 only (random intercept vs. random slopes). In order to test hypothesis 2b (prospective), models 3 and 4 contained defeat at *t* − 1 as a time-lagged predictor (random intercept vs. random slopes). Models 5 and 6 contained defeat at *t* − 1 as a time-lagged predictor as well as entrapment at *t* − 1 as a time-lagged predictor (random intercept vs. random slopes) in order to control for autoregressive effects of entrapment. To avoid between-days lags, the last value per day was not lagged. It is usually recommended to allow random slopes in models wherever possible [74]. Deviance tests were conducted for all models to assess whether random intercept or random slopes models revealed a better fit. Generally speaking, larger deviance indicates a poorer fitting model [75].

## 3. Results

### 3.1. Variability of Defeat and Entrapment (Hypotheses 1a and 1b)

#### 3.1.1. Variability of Defeat and Entrapment between Baseline and Post Assessment (Hypothesis 1a)

Regarding the question of whether feelings of defeat and entrapment differed between baseline (T0) and post (T2) assessment (Hypothesis 1a), results of *t*-tests for dependent samples and effect sizes *d* [55]—including 95% confidence intervals (CI)—are reported.

Defeat scores at T0 (*M* = 0.55, *SD* = 0.65) were significantly higher than at T2 (*M* = 0.43, *SD* = 0.56), *t*(59) = 2.025, *p* = 0.047. The effect size *d* = 0.20 (CI −0.16; 0.55) was small.

There was no significant difference between entrapment scores at T0 (*M* = 0.50, *SD* = 0.66) and T2 (*M* = 0.49, *SD* = 0.63), *t*(59) = 0.244, *p* = 0.808. The effect size *d* = 0.02 (CI −0.34; 0.37) was small.

#### 3.1.2. Variability of Defeat and Entrapment during EMA (Hypothesis 1b)

Regarding the question of whether feelings of defeat and entrapment differed during EMA (Hypothesis 1b), results of the intercept-only models for defeat and entrapment are reported. Additionally, ICCs [66] as well as MSSDs [67] are reported in order to examine the proportion of variance explained by the two different levels as well as point-to-point variability of defeat and entrapment. Inter- and intra-individual variability of defeat and entrapment over the 35 measurements is also visualized through heatmaps. Descriptive statistics and variability indices of the EMA scales and single items of defeat and entrapment used in this section can be found in Table 1.

Intercept-only models revealed grand means for entrapment and defeat of 1.17 (SD = 0.05; *t*(60) = 25.91, *p* < 0.001) and 1.15 (SD = 0.05; *t*(60) = 25.06, *p* < 0.001). Scale scores ranged between one and five, indicating that participants used the full range of answers. The proportion of nonzero ratings was 27.8% for defeat and 29.8% for entrapment. Out of all participants, 31 (50.8%) did not report any feelings of entrapment and 32 (52.5%) did not report any feelings of defeat, which, in turn, means that approximately 50% of participants reported feelings of defeat and entrapment, despite the homogeneously healthy sample.

MSSDs showed on average low prompt-to-prompt variability for defeat and entrapment. Nonetheless, MSSDs ranged from 0 to >1, indicating that variability differed between individuals (see Table 1). In fact, 29 participants (47.5%) showed no prompt-to-prompt variability in defeat and 30 participants (49.2%) showed no prompt-to-prompt variability in entrapment, which, in turn, means that more than 50% of participants showed variability in both variables. The ICCs indicate that 48% of variance accounted for within-person variability in defeat. For entrapment, 46% of variance accounted for within-person variability (see Table 1).

**Figure 3:**
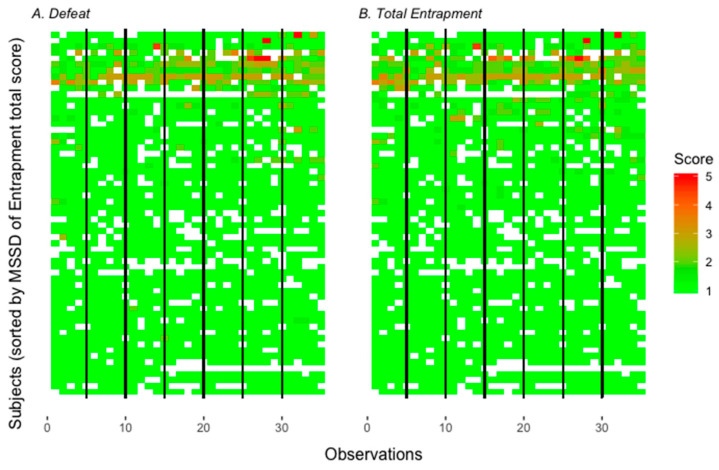
Heatmaps for momentary ratings of defeat and entrapment across observations and participants. Ratings of momentary defeat and entrapment are coded by color. Green symbolizes low momentary ratings and red symbolizes high momentary ratings of the construct. Missings are coded white. In each heatmap, one row represents one participant and one square represents one momentary rating. On the x-axis, vertical lines separate the days of the assessments (days 1–7). Additionally, numbers in steps of ten (0, 10, 20, 30) indicate the number of assessments (total 35 assessments). On the y-axis, participants are sorted by their MSSD score of total entrapment (participants with high entrapment MSSD scores are represented in the upper portion of the heatmap and participants with low entrapment MSSD scores are represented in the lower portion of the heatmap). Momentary ratings of the represented variables vary within participants and across time, as is pictured here. Overall, the lower part of the heatmap is dominated by green, and red shades are mostly missing, meaning missing or very low levels of defeat and entrapment for many participants.

Heatmaps visualize individual prompt-to-prompt variability of defeat and entrapment (see Figure 3).

### 3.2. Relationship between Defeat and Entrapment According to the IMV Model (Hypotheses 2a and 2b)

Regarding the question of whether defeat predicts entrapment cross-sectionally (at the same measurement; Hypothesis 2a as well as prospectively (from measurement to measurement; Hypothesis 2b, as stated by the IMV model [6], six models were fitted (see Section 2.3.2) and Quasi R^2^ [71] of all models as well as deviance tests between random intercept and random slopes models were calculated.

Deviance tests showed that random slopes models (M2, M4, M6) revealed a better fit (see Table 2) in comparison to their random intercept equivalents (M1, M3, M5), indicating significant differences in slopes for the association between defeat and entrapment. Therefore, only random slopes models are presented (see Table 2).

First, the relationship between defeat and entrapment was assessed cross-sectionally (at the same measurement, Hypothesis 2a. Therefore, in model 2, defeat at *t* (the same measurement) was the only predictor. In model 2, slopes between defeat at *t* and entrapment at *t* were allowed to differ between participants. Hence, in this model, both intercepts and slopes could vary between participants. When assessing cross-sectional relations between defeat and entrapment Hypothesis 2a in model 2, defeat at *t* was significantly positively associated with entrapment at *t*. For the majority of participants, the significantly differing association between defeat at *t* and entrapment at *t* in terms of slopes was positive (92.22%; see ratio of slopes >0 in Table 2). Defeat at *t* accounted for 51.81% of residual variance in entrapment at *t* (level 1), as indicated by Quasi R^2^ [66].

Second, the relationship between defeat and entrapment was assessed prospectively (from measurement to measurement, Hypothesis 2b. Therefore, in model 4, instead of defeat at *t*, defeat was added as a time-lagged predictor (at *t* − 1, the previous measurement). In model 4, slopes between defeat at *t* − 1 (the previous measurement) and entrapment at *t* (the subsequent measurement) were allowed to differ between participants. Hence, both intercepts and slopes could vary between participants in this model. When assessing the prospective prediction of entrapment (hypothesis 2b) in model 4, defeat at *t* − 1 was not significantly positively associated with entrapment at *t* (level 1). However, for the majority of participants, the significantly differing association between defeat at *t* − 1 and entrapment at *t* in terms of slopes was positive (63.68%; see ratio of slopes >0 in Table 2). In line with this result, defeat at *t* − 1 accounted for only 1.46% of residual variance in entrapment at *t* (level 1) as indicated by Quasi R^2^ [66].

Third, in addition to hypothesis 2b, we controlled for autocorrelative effects of entrapment. Therefore, in model 6, we included entrapment at *t* − 1 as a time-lagged predictor into the model in addition to the time-lagged predictor defeat. In model 6, slopes between defeat at *t* − 1 and entrapment at *t* − 1 (both at the previous measurement) and entrapment at *t* (at the subsequent measurement) were allowed to differ between participants. Hence, in this model, both intercepts and slopes could vary between participants. In model 6, when controlling for autocorrelative effects of entrapment, defeat at *t −* 1 was not significantly positively associated with entrapment at *t* as well. However, as might be expected, entrapment at *t* − 1 significantly positively predicted entrapment at *t* (from one measurement to the next). Slopes between entrapment at *t −* 1 as well as defeat at *t* − 1 and entrapment at *t* differed significantly between participants. Accordingly, for less than half of the participants, the association between defeat at *t* − 1 and entrapment at *t* was positive (44.83%), but for the majority of participants, the association between entrapment at *t* − 1 and entrapment at *t* was positive (79.95%; see ratio of slopes >0 in Table 2). Defeat at *t* − 1 and entrapment at *t* − 1 accounted for 10.2% of residual variance in entrapment at *t* (level 1) as indicated by Quasi R^2^ [66].

Last, it shall be noted that overall, model 2 revealed the best fit, as indicated by the best Quasi R^2^.

## 4. Discussion

This study aimed at investigating whether feelings of defeat and entrapment in healthy participants change over time. Additionally, we investigated the relationship between defeat and entrapment as proposed by the IMV model [6]. Two main findings of this study can be noted. First, defeat and entrapment change over time, both between as well as within participants. Second, the relationship between defeat and entrapment as proposed by the IMV model [6] could only partially be supported. While defeat and entrapment were significantly positively associated at the same measurement, defeat could not predict entrapment prospectively from measurement to measurement.

### 4.1. Variability of Defeat and Entrapment (Hypotheses 1a and 1b)

Our results only partially supported hypothesis 1a: While defeat differed significantly between baseline and post assessment, no such effect could be found for entrapment, and effects were small. However, in line with hypothesis 1b, defeat and entrapment varied between as well as within participants over a short time frame (i.e., intervals of a few hours) during EMA. Although our data revealed a low prompt-to-prompt variability in our healthy sample, we found interindividual differences between participants, with approximately half of the participants showing variability in defeat and entrapment over time. Additionally, approximately half of the participants reported defeat and entrapment scores >0 and for both defeat and entrapment, participants used the full range of answers during EMA despite the homogeneously healthy sample. Overall, findings indicate that both defeat and entrapment tend to considerably fluctuate over time.

Within the social rank theory [15], defeat and entrapment are more defined as a state than a trait. In line with Gilbert’s assumptions, we could show that both constructs are subject to significant fluctuations over time. Our findings add on to previous evidence for the variability of risk factors of suicidal ideation and behavior (e.g., [33,34,36]). Results also further underline the need to assess risk factors, such as defeat and entrapment, repeatedly and in real time in order to have a clearer perspective on their beginning and development over time.

However, one important question arising from our analyses is why defeat and entrapment only vary for approximately half of the sample and why effects between baseline and post assessment were only small. It seems possible that the variability of defeat and entrapment depends on a specific causal stimulus or situation. In this regard, some participants might just not have experienced certain situations that could be associated with or even cause heightened feelings of defeat and/or entrapment, while some other participants did. In line with this assumption, Trachsel and colleagues [60] argue that entrapment is likely to vary due to its state-like conceptualization and that it seems plausible that entrapment caused, for example, by a long-lasting, problematic relationship might be more stable over time than entrapment caused by a spontaneous short-term situation. In fact, they could prove that the stability of entrapment is lower than the stability of helplessness but higher than the stability of perceived stress. Further research on this topic, taking into account long- and short-term sociodemographic information as well as context factors, seems warranted.

### 4.2. Relationship between Defeat and Entrapment According to the IMV Model (Hypotheses 2a and 2b)

While we could confirm Hypothesis 2a and found that defeat is significantly positively associated with entrapment cross-sectionally (at the same measurement), no such effect could be found for prospective analyses in terms of defeat predicting entrapment from measurement to measurement even when controlling for autocorrelation of entrapment. Hypothesis 2b, therefore, could not be supported. This result is contrary to the assumptions of the IMV model [6], which assumes that defeat significantly positively predicts entrapment. Even though we could not find defeat predicting entrapment from measurement to measurement, further aspects should be taken into consideration.

First, our analyses did not take into account any threat to self-moderators that might moderate the relationship between defeat and entrapment according to the IMV model [6]. Maybe the inclusion of threat to self-moderators would have entailed a different outcome prospectively. Similarly, it seems possible that threat to self-moderators has a stronger influence on entrapment and, for instance, not only moderates but mediates the relationship between defeat and entrapment. It could be possible that feelings of defeat and humiliation lead to more rumination or deficits in social problem solving, which in turn could lead to entrapment. Hence, further longitudinal research including threat to self-moderators is necessary.

Second, it should be noted that many studies assessing defeat and entrapment controlled for depression (e.g., [11]) or hopelessness [76], which we did not. Although this procedure is in line with the recent argumentation by Rogers and colleagues [77], it has to be kept in mind when interpreting the current results.

Third, defeat and entrapment are two highly correlated constructs. Earlier studies even found defeat and entrapment to be better defined as a single construct [21] or as two aspects of one underlying factor [17]. Recently, Forkmann and colleagues could prove that defeat and entrapment are highly correlated yet distinct constructs [20]. In light of these aspects, it seems possible that defeat predicts entrapment very quickly (e.g., within very few minutes or seconds) due to their high correlation, and our sampling frequency was not high enough to detect this effect.

Lastly, Taylor and colleagues [21] proposed that defeat and entrapment possibly differ individually not only in their existence but also in their relationship with each other. In other words, some participants may show an association between defeat and entrapment, while others may not. In fact, in our study, random slopes models revealed a better fit. One could assume that defeat was not predictive for entrapment because some people might show less variance within feelings of defeat and entrapment than others. Further analyses including cross-level interactions analyses are necessary to further assess this topic. Promoting this idea even more, Franklin and colleagues [29] argued that in order to accurately predict suicidal ideation and behavior, there might not be one single model to fit all individuals across all populations. Pathways within the current IMV model, such as the pathway between defeat and entrapment, might not be complex enough to accurately describe differences between individuals. Constructs, such as defeat and entrapment, may interact in a more complex manner and more individually with one another than models assume. Consequently, a larger number of potential individual moderators and mediators could have an impact on the relationship between defeat and entrapment.

### 4.3. Strengths and Limitations

Most importantly, this study was the first study to apply EMA for examining defeat and entrapment. The applied method showed good feasibility. Participants showed good compliance both at the baseline and post assessments as well as during the EMA phase of the study. This study is the first to show that it is possible to assess defeat and entrapment via EMA. To our knowledge, this study is also the first to show fluctuations over time for defeat and entrapment in healthy participants with short follow-up intervals.

Despite the study’s strong points, some limitations should be taken into consideration when interpreting the current results. Although we could show considerable within-person variability for both defeat and entrapment, variance might have been reduced due to our sample consisting of healthy participants. Hence, examining defeat and entrapment in a clinical sample may reveal even higher variability in both constructs.

It is possible that our sampling scheme was not ideal to capture the frequency and amplitude of fluctuations of defeat and entrapment. In a healthy sample, these constructs might fluctuate at a low amplitude. Since the items applied in this study are usually used to detect clinically relevant feelings of defeat and entrapment, one might speculate that these items might not be sensitive enough to detect this low amplitude variation. Hence, the scaling or wording of the EMA items would have to be adjusted in order to detect smaller changes in defeat and entrapment within healthy samples. Furthermore, EMA prompts were up to two hours apart. It is possible that defeat and entrapment fluctuate at a higher frequency and, therefore, some of the possible fluctuations might not have been detected with this assessment scheme. However, these considerations are speculative, and the data do not provide any indication of this. Nevertheless, these aspects may have led to limited variance in the data, which may complicate the detection of relationships between defeat and entrapment.

## 5. Conclusions

To our knowledge, this study was the first to successfully assess feelings of defeat and entrapment in healthy participants using EMA. Not only did defeat and entrapment show considerable between—as well as within-person variability during assessments, they also proved to be significantly positively associated at the same measurement. However, contrary to the assumptions of the IMV model, defeat could not predict entrapment from measurement to measurement within our study. Defeat and entrapment have proven to be transdiagnostic, important constructs [14]. The variability we see in defeat and entrapment within EMA underlines the importance of assessing these constructs within short time frames in order to adequately depict inter- and intra-individual differences. Current results should promote the use of the EMA items applied in this study to assess defeat and entrapment in future studies and facilitate research on this topic. If similar findings to ours continue to accumulate in the future—showing that predictors of suicidal ideation fluctuate within the person over short time intervals—it should encourage considerations of potential implications for revising current theory models of suicidal ideation and behavior.

## Figures and Tables

**Table 1:** Descriptive statistics and variability indices of the ecological momentary assessment (EMA) scales of defeat and entrapment.

	*M*	*SD*	Range		*M* MSSD	*SD* MSSD	Min MSSD	Max MSSD	ICC
			Min	Max					
*Defeat*	*1.15*	*0.47*	*1*	*5*	*0.13*	*0.27*	*0*	*1.40*	*0.52*
Item 1	1.17	0.53	1	5	0.19	0.35	0	1.60	0.49
Item 2	1.13	0.47	1	5	0.13	0.28	0	1.33	0.46
*Entrapment*	*1.17*	*0.49*	*1*	*5*	*0.13*	*0.27*	*0*	*1.14*	*0.54*
Item 1	1.14	0.49	1	5	0.16	0.35	0	1.51	0.46
Item 2	1.20	0.56	1	5	0.18	0.28	0	1.45	0.52

Note. EMA = ecological momentary assessment, *M* = averaged mean scores, *SD* = standard deviation, Min = minimum value, Max = maximum value, MSSD = mean squared successive difference, ICC = intraclass correlation.

**Table 2:** Parameter estimates for multilevel models with random slopes.

Model	Fixed Effects					Random Effects		Deviance
Predictors	Est.	95% CI (Est.)	SE	*t*(df)	*p*	Slopes >0 ^1^	Variance	
*Model 2—Random Slopes*								153.97
Intercept	1.17		0.05	25.07 (60)	<0.001		0.13	
Defeat	0.52	−0.2–1.24	0.08	6.71 (60)	<0.001	92.22%	0.13	
Deviance test ^2^: *x*^2^(2) = 92.66, *p* < 0.001 Quasi R^2^: Predictors of model 2 account for 51.81% of residual variance in entrapment at level 1 ^3^								
*Model 4—Random Slopes*								965.97
Intercept	1.17		0.05	24.74 (60)	<0.001		0.13	
Defeat (*t* − 1)	0.08	−0.37–0.53	0.06	1.30 (60)	0.20	63.68%	0.05	
Deviance test ^2^: *x*^2^(2) = 80.62, *p* < 0.001 Quasi R^2^: Predictors of model 4 account for 1.46% of residual variance in entrapment at level 1 ^3^								
*Model 6—Random Slopes*								883.73
Intercept	1.17		0.05	24.73 (60)	<0.001		0.13	
Defeat (*t* − 1)	−0.02	−0.32–0.28	0.05	−0.41 (60)	0.68	44.83%	0.02	
Entrapment (*t* − 1)	0.25	−0.33–0.83	0.08	3.26 (60)	0.002	79.95%	0.09	
Deviance test ^2^: *x*^2^(2) = 137.02, *p* < 0.001 Quasi R^2^: Predictors of model 6 account for 10.2% of residual variance in entrapment at level 1 ^3^								

Notes: *n* (Level 1) = 1755. *n* (Level 2) = 61. All Level 1 predictors were person-mean-centered. Est. = estimate (unstandardized regression coefficient). 95% CI (Est.) = 95% coefficient interval for Est. SE = standard error. ^1^ Based on the assumptions of normally distributed slope coefficients, this value indicates the estimated percentage of slope coefficients that are positive [44]. For all slopes, *p* was <0.001. ^2^ Deviance tests: random intercepts vs. random slopes model (M1 vs. M2, M3 vs. M4, M5 vs. M6). ^3^ Quasi R^2^ indicates the change of the residual variance in entrapment when adding the models’ level 1 predictors compared to the baseline models [66].
